# Levothyroxine Supplementation in Euthyroid Pregnant Women With Positive Autoantibodies: A Systematic Review and Meta-Analysis

**DOI:** 10.3389/fendo.2022.759064

**Published:** 2022-02-17

**Authors:** Raffaella Di Girolamo, Marco Liberati, Claudia Silvi, Francesco D’Antonio

**Affiliations:** Center for Fetal Care and High-Risk Pregnancy, Department of Obstetrics and Gynecology, University of Chieti, Chieti, Italy

**Keywords:** thyroid disorders in pregnancy, preterm birth (PTB), thyroid disorders in IVF, thyroid autoimmune status, euthyroid pregnant women

## Abstract

**Objectives:**

To explore the role of levothyroxine (LT4) supplementation in affecting the outcome of pregnant euthyroid women with thyroperoxidase (TPO) antibodies.

**Methods:**

MEDLINE, EMBASE, Google Scholar, and the Web of Science databases were searched. The primary outcome was pre-term birth (PTB), defined as live birth before 37 weeks of gestation; secondary outcomes were gestational hypertension, pre-eclampsia (PE), placental abruption, miscarriage, intra-uterine death (IUD), and admission to neonatal intensive care unit (NICU). All these outcomes were explored in euthyroid women with TPO antibodies receiving compared to those not receiving LT4 supplementation in pregnancy. Random-effect meta-analyses were used to analyze the data and results reported as pooled odds ratios (OR) with their 95% confidence intervals (CI).

**Results:**

The risk of PTB was lower in women with TPO antibodies receiving compared to those not receiving LT4 supplementation (OR of 0.60 (95% CI 0.4-0.9). However, this association came mainly from observational studies (OR: 0.29, 95% CI 0.1-0), while RCTs did not show any beneficial effect of LT4 supplementation in affecting such outcomes. Conversely, there was no difference in the risk of gestational hypertension, preeclampsia, placental abruption, miscarriage, and admission to NICU between the two groups.

**Conclusions:**

LT4 supplementation in TPO euthyroid women is not associated with a reduced risk of PTB in TPO-positive women with normal thyroid function.

## Introduction

Thyroid diseases affect up to 4% of all pregnancies with primary hypothyroidism being the most prevalent disease (1). Inadequately treated or subclinical hypothyroidism increases are associated with an increased risk of adverse pregnancy outcomes, including placental abruption, preterm birth, miscarriage, preeclampsia, gestational diabetes, and sub-optimal neurocognitive development (2, 3). However, not all studies have found an increased risk of adverse outcomes with hypothyroidism and the pathophysiology of the association between altered thyroid function and adverse pregnancy outcome has not been completely elucidated yet. Despite that, American Thyroid Association recommends universal treatment of maternal hypothyroidism, irrespective of antibody status (4). More recently, several observational studies reported that thyroperoxidase antibody (TPOAb)-positive women with a normal thyroid function are at also increased risk of experiencing adverse pregnancy outcomes (5). However, there is still insufficient whether levothyroxine (LT4) supplementation may improve pregnancy outcomes in these women (6, 7) The aim of this systematic review was to explore the role of LT4 supplementation in affecting maternal and perinatal outcomes in euthyroid women with TPO antibodies.

## Methods

### Protocol, Information Sources, and Literature Search

This systematic review was performed according to an *a priori* protocol recommended for systematic reviews and meta-analysis. PRISMA guidelines were followed (8).

MEDLINE, EMBASE, Google Scholar, and the Web of Science databases were searched electronically up to August 14, 2021, using the following search terms (as words in the title/abstract), and combinations of the relevant medical subject heading (MeSH) terms, keywords, and word variants for “Euthyroid women”, “LT4 supplementation”, “Perinatal outcome” and “Maternal outcome”. Studies including euthyroid pregnant women with thyroid autoantibodies treated compared to those not treated with levothyroxine were included. The search and selection criteria were restricted to the English language. Reference lists of relevant articles and reviews were hand-searched for additional reports.

### Outcomes Measures, Study Selection, and Data Collection

The primary outcome was pre-term birth (PTB), defines as birth before 37 weeks of gestation.

The secondary outcomes were:

- Gestational hypertension, defined as blood pressure ≥140/90 mmHg
- Pre-eclampsia (PE), defined as gestational hypertension accompanied by one or more of the following new-onset conditions at or after 20 weeks gestation: proteinuria, other maternal organ dysfunction, including acute kidney injury, liver involvement, neurological or hematological complications, or uteroplacental dysfunction (such as fetal growth restriction, abnormal umbilical artery Doppler waveform analysis, or stillbirth)
- Placental abruption, defined as the separation of the placenta from the wall of the uterus during pregnancy
- Miscarriage, defined as the loss of an embryo or fetus before the 20^th^ week of pregnancy
- Intra-uterine death, defined as a fetal death detected at ≥24 weeks
- Admission to neonatal intensive care unit (NICU)

All these outcomes were explored in the euthyroid women with TPO antibodies receiving compared to those not receiving LT4 supplementation in pregnancy.

Two authors (RDG, FDA) reviewed all abstracts independently. Agreement regarding potential relevance was reached by consensus. Full-text copies of those papers were obtained, and the same two reviewers independently extracted relevant data regarding study characteristics and maternal and perinatal outcomes with LT4 supplementation. Inconsistencies were discussed by the reviewers and consensus was reached by discussion with the senior authors (FDA).

### Risk of Bias and Statistical Analysis

Quality assessment of the included studies was assessed using the Revised Cochrane risk-of-bias tool for randomized trials (RoB 2) (9). According to this tool, the risk of bias of each included study is judged according to five domains: bias arising from the randomization process, bias due to deviations from intended interventions, bias due to missing outcome data, bias in the measurement of the outcome, and bias in the selection of the reported result. Although the RoB 2 tool does not provide an overall risk of bias assessment, the overall risk of bias was considered low if four or more domains were rated as low risk (not counting ‘other biases’), with at least one of them being sequence generation or allocation concealment, according to what reported in previous systematic reviews of intervention. Finally, the quality of evidence and strength of recommendations were assessed using the Grading of Recommendations Assessment, Development, and Evaluation (GRADE) methodology **(**GRADE pro, Version 20. McMaster University, 2014) (10).

Conversely, quality assessment for non-randomized trials was assessed using the Newcastle-Ottawa Scale (NOS) for case-control studies. According to the NOS, each study is judged on three broad perspectives: the selection of the study groups, the comparability of the groups, and the ascertainment outcome of interest. The assessment of the selection of a study includes the evaluation of the representativeness of the exposed cohort, selection of the non-exposed cohort, ascertainment of exposure, and the demonstration that the outcome of interest was not present at the start of the study. The assessment of the comparability of the study includes the evaluation of the comparability of cohorts based on the design or analysis. Finally, the ascertainment of the outcome of interest includes the evaluation of the type of the assessment of the outcome of interest and the length and the adequacy of follow-up. According to the NOS, a study can be awarded a maximum of one star for each numbered item within the Selection and Outcome categories. A maximum of two stars can be given for Comparability (11).

Small study effects (potentially caused by publication bias) were assessed using funnel plots and formally tested through the Egger regression asymmetry test for those meta-analyses including ≥10 studies. When less than 10 studies are included, the available tests are at a very high risk of bias because of the lack of statistical power (12).

Head-to-head meta-analyses using the random-effect model were used to analyze the data and results reported as pooled odds ratios (OR) with their 95% confidence intervals (CI). RevMan 5.3 (Copenhagen: The Nordic Cochrane Centre, The Cochrane Collaboration, 2014) Stata, version 13.1 (Stata Corp., College Station, TX, 2013) and Comprehensive Meta-analysis (Biostat, Englewood NJ, 07361) were used to analyze the data.

## Results

### Study Selection and Characteristics

**Figure 1:**
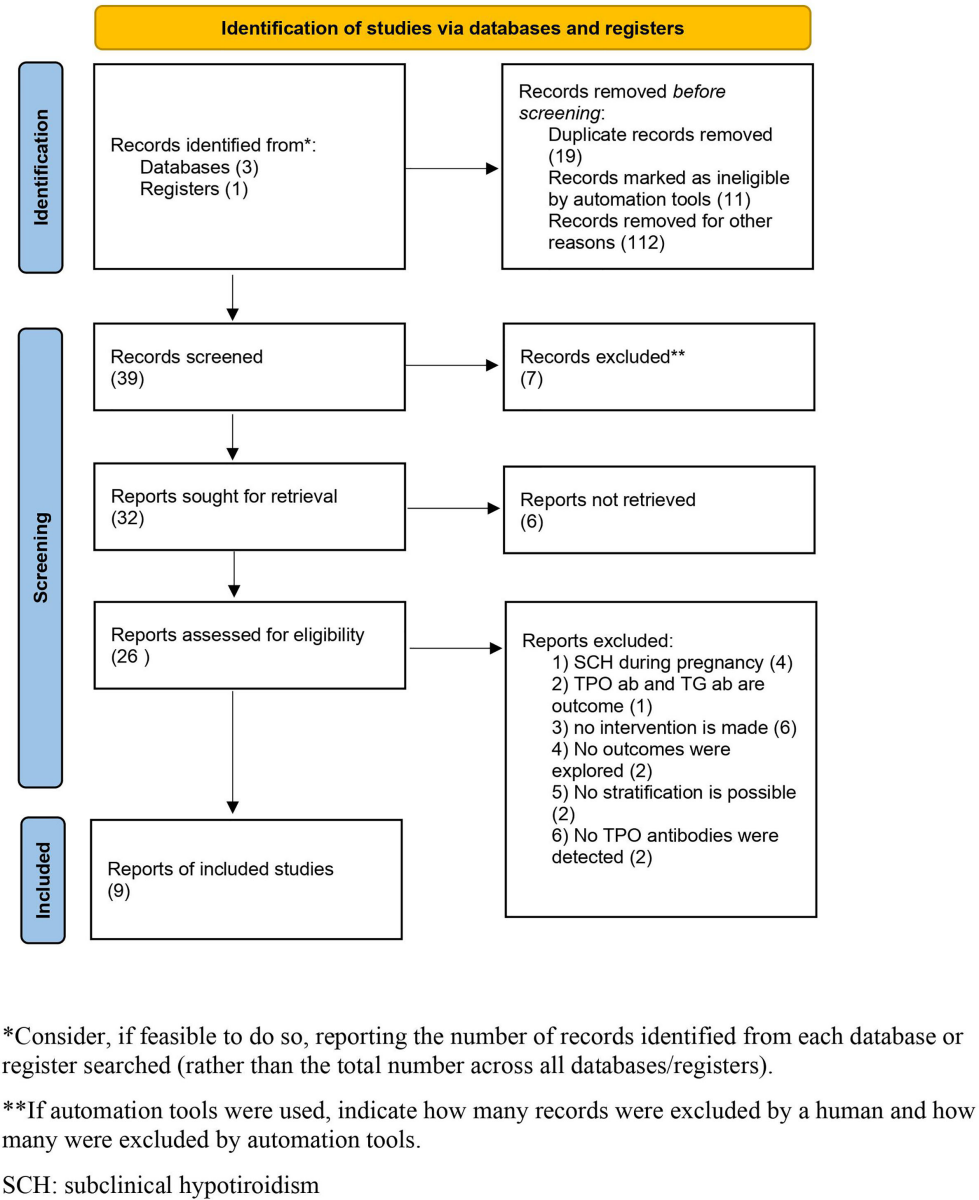
Flow diagram of the systematic review.

There were 39 articles initially selected, of which 26 were assessed with respect to their eligibility for inclusion and 9 (2, 3, 5, 13–18) were included in the systematic review (**Supplementary Table 1**). There were 4 randomized clinical trials (RCTs), 3 were prospective, and 2 were retrospective series (**Table 1**, **Figure 1**). When considering the RCTs, the studies by Dhillon-Smith et al. (14) and Wang et al. (15) explored the role of LT4 supplementation before conception in women undergoing *in vitro* fertilization and in those with a history of miscarriage and infertility, respectively, while those by Nigro et al. (16) and Narzapour et al. (5) evaluated obstetric complications in pregnancy as their primary outcome.

**Table 1:** General characteristics of the included studies.

First Author	Year	Study Design	Period Considered	Outcomes Observed (Maternal)	Outcomes Observed (Fetal)	Reference Values For Thyroid Status*	Starting Dose LT4 Supplementation (µg/d)	Women (n)
Dhillon-Smith et al. (14)	2019	RCT	NS	Miscarriage, preterm birth, clinical pregnancy rate and live birth	None	TSH: (0.44-3.63) FT4: (0.0-21.0) **	50	266
Narzapour et al. (5)	2017	RCT	NS	Placental abruption, Miscarriage, Preterm birth	Admission to NICU, Stillbirth, Birthweight	TSH (0.1–2.5): FT4: (1–4.5) TPO: (<50)	0.5 μg/kg/d (TSH <1.0 μIU/mL) 0.75 μg/kg/d (TSH 1.0–2.0 μIU/mL) 1 μg/kg/d (TSH >2.0 μIU/mL)	131
Wang et al. (15)	2017	RCT	2012-2016	Miscarriage, preterm birth, clinical pregnancy rate and live birth	None	TSH: (0.5-4.78) TPOAb: (<60)	50 (TSH ≥2.5mIU/L) 25 (If TSH <2.5 mIU/L)	282
Stoian et al. (2)	2016	Prospective cohort	NS	Spontaneous miscarriage	APGAR score, Week of Gestation; Birth Length (cm); Birthweight (kg)	TSH: (>2.5)	25 (TSH ≥4.5mIU/L) 12.5 (If TSH <4.5 mIU/L)	107
Negro et al. (16)	2016	RCT	2011-2014	Miscarriage, preterm birth	None	TSH: (0.5-2.5) TPOAb: (≤16)	0.5 μg/kg/d (TSH 0.5-1.5 μIU/mL) 1 μg/kg/d (TSH 1.5–2.5 μIU/mL)	198
Lata et al. (13)	2013	Prospective cohort	2010-2011	Miscarriage, Preterm labor (PTL), Gestational hypertension, Preterm premature rupture of membranes, Intrauterine growth retardation	None	TSH: (0.27–4.2) FT3: (1.7–4.2) FT4: (0.7–1.8) anti-TPO: (<34)	25	31
Lepoutre et al. (18)	2012	Retrospective	2008-2009	Miscarriage, preterm birth and placental abruption	None	TSH: (0.2–3.5) FT4: (0.6–1.4) TPO-Ab: (<9)	NS: the initial LT4 dose started as soon as TPOAb was detected and TSH >1 mU/l	49
Revelli et al. (17)	2009	Retrospective	2004-2008	Miscarriage, pregnancy rate, ongoing pregnancy rate	None	TPO-Ab: (0–40) Tg-Ab: (0–35)	50	55
Negro et al. (3)	2006	Prospective cohort	2002-2004	Hypertension, Preeclampsia, Placental abruption, Miscarriage, Preterm birth	None	TSH: (0.27–4.2) FT4: (9.3–18.0) TPO-Ab: (<100)	0.5 μg/kg/d (TSH <1.0 μIU/mL) 0.75 μg/kg/d (TSH 1.0–2.0 μIU/mL) 1 μg/kg/d (TSH >2.0 μIU/mL)	115

NS, not specified; RCT, Randomized Controlled Trial. *Values are expressed in: 1. IU/L for TPO and Tg antibodies. 2. mIU/L for TSH. 3. ng/ml for FT3 and FT4. **Value expressed in mol/L.

Overall, these nine studies included 1884 pregnant TPO Ab positive euthyroid women, aged between 16 and 41 [mean ± standard deviation (SD)=30.75 ± 4.56]; out of these, 935 were treated with LT4 supplementation. TSH levels were 2.5 ± 0.5 and 2.2 ± 0.5 in women treated compared to those not treated with LT4 in RCTs, while the corresponding figures for observational studies were 3.2 ± 1.6 and 1.19 ± 1.2, respectively.

Assessment of the RCTs included in the present systematic review using GRADE showed a low quality of evidence for the primary outcome.

The results of the quality assessment of the included studies using the NOS for observational studies are presented in **Supplementary Table 2**. The included studies showed an overall good score regarding the selection and comparability of the study groups, and for ascertainment of the outcome of interest. The main weaknesses of these studies were their non-randomized design, small sample size, and heterogeneity in outcome definition and assessment.

### Synthesis of the Results

When considering RCTs and observational studies together, the risk of PTB was lower in TPO-positive women with normal thyroid function receiving compared to those not receiving LT4 supplementation with an OR of 0.60 (95% CI 0.4-0.9; I^2^: 53.9%) (**Table 2**). Likewise, the risk of admission to NICU was lower in these women (OR: 0.14, 95% CI 0.03-0.7; I^2^: 0%), while there was no difference in the risk of gestational hypertension (p=0.23), preeclampsia (p=0.48), placental abruption (p=0.11), miscarriage (p=0.37), between the two groups.

**Table 2:** Pooled Odds ratio (OR) for the different categorical outcome explored in the present systematic review in euthyroid women having compared to those not having LT4 supplementation.

Outcome	Studies	Pregnancies	Pooled OR (95% CI)	I^2^ (%)	p-value (*P*<0.05)
			***All studies***
**Pre-term birth**	6	59/871vs 93/903	0.60 (0.4-0.9)	53.9	**0.008**
**Gestational Hypertension**	3	8/137 vs 13/154	0.56 (0.2-1.4)	0	0.23
**Pre-eclampsia**	2	4/106 vs 5/85	0.61 (0.1-2.3)	0	0.48
**Placenta abruption**	3	1/171 vs 1/151	0.85 (0.08-8.5)	7,7	0.11
**Miscarriage**	9	121/935 vs 142/949	0.82 (0.5-1.2)	28.1	0.37
**Intra-uterine death**	3	1/438 vs 0/449	3.11 (0.1-76.5)	0	0.488
**Admission to NICU**	2	2/163 vs 12/167	0.14 (0.03-0.7)	0	**<0.001**
			***Randomized controlled trials***
**Pre-term birth**	3	50/627 vs 65/640	0.74 (0.4-1.3)	50.2	0.311
**Gestational Hypertension**	0	–	–	–	–
**Pre-eclampsia**	0	–	–	–	–
**Placenta abruption**	1	0/65 vs 0/66	1.02 (0.01-51.9)	–	0.994
**Miscarriage**	4	112/636 vs 126/648	0.88 (0.7-1.2)	0	0.999
**Intra-uterine death**	1	1/266 vs 0/274	3.10 (0.1-76.5)	–	0.489
**Admission to NICU**	1	2/56 vs 12/58	0.14 (0.03-0.7)	–	**0.013**
			***Observational studies***
**Pre-term birth**	3	9/244 vs 28/263	0.29 (0.1-0.6)	37.3	***0.002***
**Gestational Hypertension**	3	8/137 vs 13/154	0.57 (0.2-1.4)	0	0.233
**Pre-eclampsia**	2	4/106 vs 5/85	0.62 (0.2-2.4)	0	0.483
**Placenta abruption**	2	1/106 vs 1/85	0.85 (0.01-8.6)	7.7	0.892
**Miscarriage**	5	9/299 vs 16/301	0.72 (0.1-3.6)	60	0.692
**Intra-uterine death**	1	0/107 vs 0/109	1.02 (0.02-51.8)	–	0.993
**Admission to NICU**	1	0/107 vs 0/109	1.02 (0.02-51.8)	–	0.993

NICU, neonatal intensive care unit. Bold values are statistically significant.

When considering only RCTs, there was no difference in the risk of PTB (p= 0.311), placental abruption (p= 0.994), miscarriage (p= 0.999), or IUD (p= 0.489) between TPO-positive women treated compared to those not treated with LT4, while the risk of gestational hypertension and PE was not assessed in any of the included RCT. The risk of admission to NICU was lower in TPO-positive women treated with LT4 supplementation, but this outcome was assessed only in one trial.

The risk of PTB was lower in TPO-positive women undergoing LT4 supplementation when considering only observational studies (OR: 0.29, 95% CI 0.1-0.6; I^2^: 37.3%), while there was no difference in any of the other outcomes explored in the present systematic review.

## Discussion

### Summary of the Main Findings

The findings from this systematic review show that, in TPO-positive women with normal thyroid function, LT4 supplementation does not significantly affect pregnancy and perinatal outcomes. The reduced risk of PTB observed in this systematic review came mainly from observational studies, while RCTs did not demonstrate any beneficial effect of LT4 supplementation in these women. However, not all the RCTs included in the present systematic review were specifically designed to report the effect of LT4 supplementation in affecting the primary outcome, thus limiting the clinical applicability of these findings, and highlighting the need for appropriately designed and powered studies.

### Strengths and Limitations

Thorough literature search, multitude of outcomes explored and stratification of the analysis according to the type of study (RCTs and observational studies) are the main strengths of the present systematic review.

The small number of included studies for some of the observed outcomes, dissimilarity in inclusion criteria, outcome definition and gestational age at treatment are the main weakness of the present review. Furthermore, we could not perform comprehensive sub-group analyses considering only women with specific risk factors for PTB, type of PTB and IVF status, this is likely to represent a major bias. Finally, not all the included RCTs were specifically designed to assess the role of LT4 supplementation during pregnancy, with two of them rather exploring the reproductive outcomes of TPO-positive women with normal thyroid function undergoing IVF. In this scenario, it is likely that this systematic review might be underpowered for some of the outcomes explored.

### Interpretation of the Study Findings, Clinical and Research Implications

Thyroid disorders are among the most common medical complications occurring in pregnancy. Previous systematic reviews reported a higher risk of obstetric complications in women with thyroid autoimmunity and sub-clinical hypo- or hyper-thyroidism. However, there is still conflicting evidence on whether these women should receive LT4 supplementation.

In the systematic review by Rao et al. (6), the authors reported a beneficial effect of LT4 supplementation in affecting pregnancy loss and pre-term birth rate in women with sub-clinical hypothyroidism (SCH) and thyroid auto-immune (TAI) status. Conversely, Sun et al. (19) did not report any efficacy of LT4 supplementation in affecting pregnancy and perinatal outcome in euthyroid women with thyroid autoimmunity.

In the present systematic review, we could not find any beneficial role of LT4 supplementation in affecting pregnancy and perinatal outcomes improvement TPO-positive women with normal thyroid function. This lack of association between LT4 supplementation and reduced risk of adverse pregnancy outcome, mainly PTB, can be explained on different basis. First, the included trials were not specifically designed to elucidate the role of LT4 supplementation in affecting PTB. Furthermore, the large majority of included cases were IVF pregnancies, which have been reported to be at higher risk of PTB irrespective of thyroid status. Finally, it was not possible to extrapolate whether the included cases had other risk factors for PTB.

## Conclusion

LT4 supplementation in TPO euthyroid women does not affect pregnancy and perinatal outcomes in TPO-positive women with normal thyroid function. However, the small number of included cases and dissimilarity in inclusion criteria, therapeutic strategies, and populations analyzed highlights the need for properly designed RCTs aimed at elucidating whether treatment with LT4 may reduce the risk of adverse obstetric outcomes in these women.

## Data Availability Statement

The original contributions presented in the study are included in the article/**Supplementary Material**. Further inquiries can be directed to the corresponding author.

## Author Contributions

RDG and FD’A contributed to the conception and design of the study. RDG and CS reviewed the literature and extracted the data from the article. ML and FD’A performed data analysis. All authors participated to the drafting of the article and critically revised the work.

## Conflict of Interest

The authors declare that the research was conducted in the absence of any commercial or financial relationships that could be construed as a potential conflict of interest.

## Publisher’s Note

All claims expressed in this article are solely those of the authors and do not necessarily represent those of their affiliated organizations, or those of the publisher, the editors and the reviewers. Any product that may be evaluated in this article, or claim that may be made by its manufacturer, is not guaranteed or endorsed by the publisher.

## Abbreviations

COS, controlled ovarian stimulation; GDM, gestational diabetes mellitus; IVF, *in vitro* fertilization; LT4, levothyroxine; LT4-pregnant, women treated with levothyroxine; (n)LT4-pregnant, women not treated with levothyroxine; RCT, randomized clinical trial; TgAb, thyroglobulin antibody; TPOAb, thyroid peroxidase antibody; SCH, sub-clinical hypothyroidism; TAI, thyroid auto-immune.
