# Supplementary material for: Levothyroxine Supplementation in Euthyroid Pregnant Women With Positive Autoantibodies: A Systematic Review and Meta-Analysis
Source: Front Endocrinol (Lausanne). 2022 Feb 17;13:759064. doi: 10.3389/fendo.2022.759064 (PMC8892207; doi:10.3389/fendo.2022.759064)
Supplement: Supplementary file 1 [file Table_1.docx]

| **Authors** | **Year** | **Title** | **reasons' exclusion** |
| --- | --- | --- | --- |
| Yuan et al | 2020 | ﻿Relationship between anti-thyroid peroxidase antibody positivity and pregnancy-related and fetal outcomes in Euthyroid women: a single-center cohort study | no intervention |
| Zhang et al | 2019 | ﻿The impact of thyroid function and TPOAb in the first trimester on pregnancy outcomes: a retrospective study in Peking | No intervention. A sample of 266 women was recruited to estimate the effect of LT4 supplement on pregnancy outcome; no stratification about TSH was made. |
| ﻿Nazarpour et al | 2018 | ﻿Effects of Levothyroxine on Pregnant Women With Subclinical Hypothyroidism, Negative for Thyroid Peroxidase Antibodies | ﻿366 pregnant women with SCH, negative for TPO-Ab |
| Maraka et al | 2017 | ﻿hyroid hormone treatment among pregnant women with subclinical hypothyroidism: US national assessment | women with SCH (TSH 2.5-10 mUI/ml) |
| Rajput et al | 2017 | Prevalence of Thyroid Peroxidase Antibody and Pregnancy Outcome in Euthyroid Autoimmune Positive Pregnant Women from a Tertiary Care Center in Haryana | no intervention |
| Maraka et al | 2016 | ﻿Effect of levothyroxine treatment on in vitro fertilization and pregnancy outcome in infertile women with subclinical hypothyroidism undergoing in vitro fertilization/intracytoplasmic sperm injection | SCH during pregnancy was defined as serum TSH >2.5 mIU/L for the 1st trimester or >3 mIU/L for the 2nd and 3rd trimesters, but >10mIU/L. |
| Maraka et al | 2016 | ﻿Subclinical Hypothyroidism in Pregnancy: A Systematic Review and Meta-Analysis | women with SCH (TSH 2.5-10 mUI/ml |
| Chen et al | 2014 | Associations between thyroid autoantibody status and abnormal pregnancy outcomes in euthyroid women | no intervention |
| ﻿Korevaar et al | 2013 | ﻿Hypothyroxinemia and TPO-Antibody Positivity Are Risk Factors for Premature Delivery: The Generation R Study | no intervention |
| Wang et al | 2012 | ﻿Effects of maternal subclinical hypothyroidism on obstetrical outcomes during early pregnancy | no TPO antobodies were detected |
| Kim et al | 2011 | ﻿Effect of levothyroxine treatment on in vitro fertilization and pregnancy outcome in infertile women with subclinical hypothyroidism undergoing in vitro fertilization/intracytoplasmic sperm injection | TPO ab and TG ab are outcome |
| Negro et al | 2011 | ﻿Thyroid Antibody Positivity in the First Trimester of Pregnancy Is Associated with Negative Pregnancy Outcomes | no intervention |
| ﻿Abbassi-Ghanavati et al | 2010 | ﻿Pregnancy Outcomes in Women With Thyroid Peroxidase Antibodies | Frequency of euthyroid women, subclinical hypothiroidism or isolated hypotyroxinemia was shown. No stratification of pregnancy outcome bethween each grupo is possible. |
| Debieve et al | 2009 | ﻿To Treat or Not to Treat Euthyroid Autoimmune Disorder during Pregnancy? | No pregnancy outcome explored |
| Mannisto et al | 2009 | ﻿Perinatal Outcome of Children Born to Mothers with Thyroid Dysfunction or Antibodies: A Prospective Population-Based Cohort Study | No stratification possible |
| ﻿Abdel Rahman et al | 2009 | ﻿IMPROVED IN VITRO FERTILIZATION OUTCOMES AFTER TREATMENT OF SUBCLINICAL HYPOTHYROIDISM IN INFERTILE WOMEN | no TPO antobodies were detected |
| Negro et al | 2005 | ﻿Levothyroxine treatment in thyroid peroxidase antibody-positive women undergoing assisted reproduction technologies: a prospective study | no pregnancy outcome explored |
