# Supplementary material for: Levothyroxine Supplementation in Euthyroid Pregnant Women With Positive Autoantibodies: A Systematic Review and Meta-Analysis
Source: Front Endocrinol (Lausanne). 2022 Feb 17;13:759064. doi: 10.3389/fendo.2022.759064 (PMC8892207; doi:10.3389/fendo.2022.759064)
Supplement: Supplementary file 2 [file Table_2.docx]

**Supplementary Table 1**. Quality assessment of the included studies according to Newcastle-Ottawa Scale (NOS) for case control-studies; a study can be awarded a maximum of one star for each numbered item within the Selection and Outcome categories. A maximum of two stars can be given for Comparability.

| **First Author** | **Year** | **Selection** | **Comparability** | **Outcome** |
| --- | --- | --- | --- | --- |
| Stoian | 2016 | ★ | ★ | ★★ |
| Lata | 2013 | ★★ | ★ | ★ |
| Lepoutre | 2012 | ★ | ★ | ★ |
| ﻿Revelli | 2009 | ★ | ★ | ★ |
| Negro | 2006 | ★★ | ★ | ★★ |
